# Case Report: Familial *TBX4*-related developmental lung disease: two siblings with fatal neonatal respiratory failure

**DOI:** 10.3389/fped.2026.1816836

**Published:** 2026-10-22

**Authors:** Ranga Prasanth Thiruvenkataramani, Tarek Mohamed, Mary v Pugmire, Said A. Omar

**Affiliations:** 1 Division of Neonatology, Department of Pediatrics, University of Michigan Health-Sparrow, Lansing, MI, United States; 2 Division of Neonatology, Department of Pediatrics and Human Development, Michigan State University, East Lansing, MI, United States

**Keywords:** congenital alveolar dysplasia, developmental lung disease, genetic counseling, neonatal respiratory failure, pulmonary hypertension, pulmonary hypoplasia, *TBX4*

## Abstract

**Background:**

T-box transcription factor 4 (*TBX4*) is a critical regulator of lung and pulmonary vascular development. Pathogenic *TBX4* variants are associated with developmental lung disease, pulmonary hypertension, and neonatal respiratory failure.

**Case presentation:**

We report two siblings with lethal neonatal respiratory failure. The first, a term female infant, died at 11 h of life, and her late-preterm male sibling died at 9 h despite maximal intensive care. Both developed severe hypoxemia and pulmonary hypertension. Autopsy demonstrated marked pulmonary hypoplasia. Whole-exome sequencing identified a novel heterozygous *TBX4* variant, c.741G>C (p.Lys247Asn), inherited from their unaffected father. Preimplantation genetic testing detected the variant in seven of 10 embryos.

**Conclusion:**

These cases expand the spectrum of *TBX4*-associated developmental lung disease and support consideration of *TBX4* testing in neonates with unexplained respiratory failure and pulmonary hypertension.

## Introduction

Developmental lung disorders are a heterogeneous group of rare congenital conditions characterized by abnormal pulmonary morphogenesis and impaired development of the airways, alveoli, and pulmonary vasculature (1–4). Severe forms, including acinar dysplasia and congenital alveolar dysplasia, often present with refractory neonatal respiratory failure, persistent pulmonary hypertension, and high mortality. Pulmonary hypoplasia (PH), characterized by reduced lung volume and impaired distal airspace development, may occur as an isolated abnormality or secondary to congenital diaphragmatic hernia, oligohydramnios, fetal thoracic compression, or neuromuscular disorders (2, 3). Increasing evidence indicates that some apparently isolated cases result from pathogenic variants affecting genes involved in fetal lung morphogenesis.

*TBX4* encodes a T-box transcription factor that plays a critical role in embryonic lung branching morphogenesis, distal airway formation, mesenchymal differentiation, and pulmonary vascular development (5, 6). Pathogenic *TBX4* variants are associated with a broad phenotypic spectrum, ranging from childhood pulmonary arterial hypertension (PAH) and small patella syndrome to severe neonatal developmental lung disease, including acinar and congenital alveolar dysplasia (7–13). Recognition of *TBX4*-related lung disorders has improved our understanding of previously unexplained neonatal respiratory failure (14–16).

We report two siblings with recurrent lethal neonatal developmental lung disease associated with a likely pathological heterozygous *TBX4* variant inherited from their clinically unaffected father. These cases expand the phenotypic spectrum of *TBX4*-associated disorders and highlight the challenges of diagnosis, counseling, and reproductive planning in families affected by incompletely penetrant developmental lung disease.

### Case 1

In 2017, a full-term (41 weeks’ gestation), appropriate-for-gestational-age female neonate weighing 3,030 g was delivered via spontaneous vaginal delivery to a 29-year-old primigravida following induction for post-term pregnancy. Maternal history was notable only for restless leg syndrome, and medications included prenatal vitamins, ranitidine, and carbidopa-levodopa. There was no known family history of neonatal respiratory failure, pulmonary hypoplasia, pulmonary hypertension, skeletal abnormalities, recurrent pregnancy loss, or congenital anomalies. Prenatal screening and serial fetal ultrasonography revealed no structural abnormalities. A fetal ultrasound performed 2 days before delivery demonstrated normal amniotic fluid volume, with an amniotic fluid index (AFI) of 9.2 cm (25th percentile). Umbilical artery Doppler studies demonstrated preserved end-diastolic flow. Measurements of thoracic circumference, abdominal circumference, thoracic-to-abdominal circumference ratio, and detailed biophysical profile (BPP) parameters were unavailable. Maternal serological screening was negative, and there was no history of oligohydramnios, fetal growth restriction, or other pregnancy complications.

The infant's Apgar scores were 7, 1, 4, 6, and 6 at 1, 5, 10, 15, and 20 min, respectively. Although initially vigorous after birth, she developed secondary apnea at approximately 4 min of life, requiring positive-pressure ventilation followed by continuous positive airway pressure (CPAP). She was subsequently admitted to the neonatal intensive care unit (NICU) with persistent hypoxemia, maintaining oxygen saturations in the mid-60% range despite CPAP at 5 cm H_2_O and an FiO_2_ of 1.0. Initial arterial blood gas demonstrated severe mixed respiratory and metabolic acidosis (pH 6.84, PaCO_2_ 97 mmHg, PaO_2_ 35 mmHg, HCO_3_ 16.9 mmol/L, base excess −23.1 mmol/L). Along with moderate-to-severe encephalopathy on modified Sarnat examination, these findings raised concern for hypoxic-ischemic encephalopathy, prompting whole-body therapeutic hypothermia.

Chest radiography (Figure 1) demonstrated markedly reduced lung volumes with diffuse bilateral pulmonary opacities, prominent air bronchograms, and a small right subpulmonic pneumothorax. The infant was intubated, administered exogenous surfactant, and transitioned to high-frequency oscillatory ventilation because of severe hypoxemic and hypercapnic respiratory failure. Initial echocardiography demonstrated structurally normal cardiac anatomy with severely depressed biventricular systolic function. Assessment of pulmonary hypertension was limited by ventricular dysfunction; however, right-to-left shunting across fetal circulatory pathways together with profound refractory hypoxemia raised strong suspicion for persistent pulmonary hypertension of the newborn (PPHN). Inhaled nitric oxide (iNO) therapy was initiated without significant clinical improvement. The clinical course was further complicated by bilateral pneumothorax and pneumomediastinum requiring needle decompression and chest tube placement.

**Figure 1:**
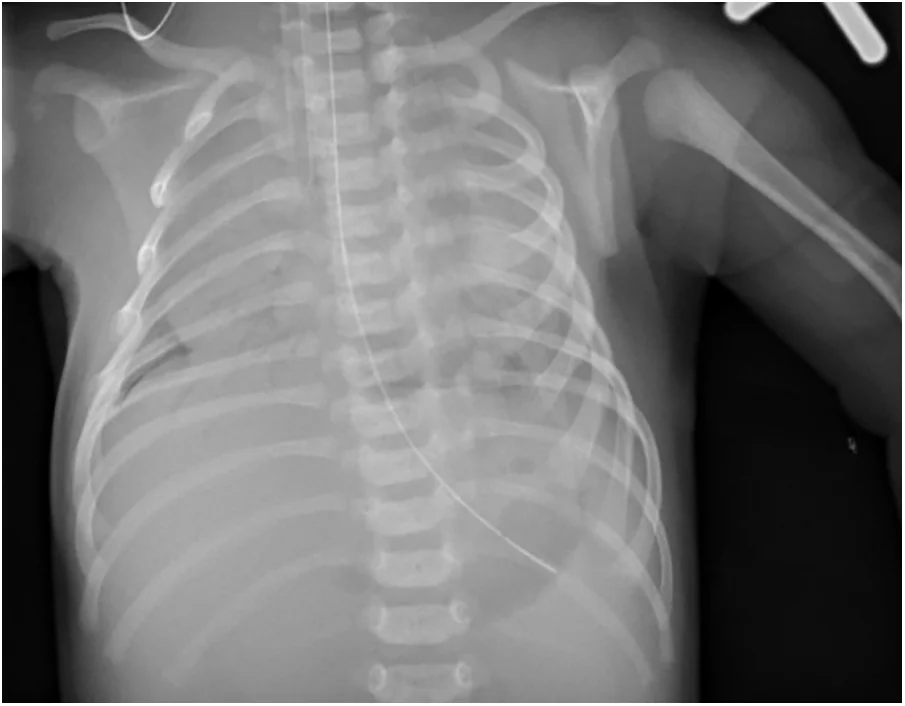
Initial chest radiograph of Case 1 demonstrates bilateral pulmonary infiltrates with air bronchograms, low lung volumes, and a small right subpulmonic pneumothorax.

Despite maximal ventilatory support and aggressive resuscitative measures, she developed disseminated intravascular coagulation and progressive cardiorespiratory deterioration culminating in cardiopulmonary arrest. Following 10 min of unsuccessful resuscitation and discussions regarding the grave prognosis, life-sustaining treatment was withdrawn, and the infant died at 11 h of life.

### Case 2

In 2019, the mother of the previously described infant delivered a late-preterm (36 weeks’ gestation), appropriate-for-gestational-age male neonate weighing 2,930 g via spontaneous vaginal delivery. Prenatal care was adequate, and no prenatal genetic testing was performed. Antenatal screening and serial fetal ultrasonography revealed no structural abnormalities. A fetal ultrasound performed approximately 1 month before delivery demonstrated normal amniotic fluid volume, with an AFI of 17 cm (62nd percentile). The fetal BPP score was 8/8. The infant’s thoracic circumference measured 22.1 cm and his abdominal circumference 27.94 cm, resulting in a thoracic-to-abdominal circumference ratio of 0.79. Umbilical artery Doppler studies demonstrated preserved end-diastolic flow. No findings suggestive of pulmonary hypoplasia or other structural anomalies were identified prenatally. Maternal serologies were unchanged from the previous pregnancy and were unremarkable. The previous pregnancy was complicated by neonatal demise secondary to pulmonary hypoplasia, pulmonary hypertension, and respiratory failure. Maternal medications included prenatal vitamins, vitamin D supplementation, and citalopram. Spontaneous rupture of membranes occurred 2 h before delivery with clear amniotic fluid.

Apgar scores were 7, 2, and 4 at 1, 5, and 10 min, respectively. The infant had a weak cry and moderate tone at birth but subsequently developed secondary apnea requiring positive-pressure ventilation. Because of an inadequate response, advanced neonatal resuscitation was initiated, including endotracheal intubation, chest compressions, and epinephrine administration via both the endotracheal tube and a low-lying umbilical venous catheter. He was transferred to the NICU at 18 min of life with persistent severe hypoxemia, maintaining oxygen saturations in the 60% range despite mechanical ventilation with an FiO_2_ of 1.0. Initial arterial blood gas analysis demonstrated severe mixed respiratory and metabolic acidosis (pH 6.87, PaCO_2_ 119 mmHg, PaO_2_ 23 mmHg, HCO_3_ 20.6 mmol/L, base excess −19.5 mmol/L). Together with findings consistent with moderate encephalopathy on modified Sarnat examination, there was concern for hypoxic-ischemic encephalopathy, and whole-body therapeutic hypothermia was initiated.

Initial chest radiography (Figure 2) demonstrated markedly low lung volumes and diffuse poor aeration, raising concern for severe developmental lung disease. The infant was transitioned to high-frequency oscillatory ventilation, administered exogenous surfactant, and required escalating ventilatory support because of refractory hypoxemic and hypercapnic respiratory failure. His course was further complicated by bilateral pneumothorax requiring chest tube placement. Cranial and renal ultrasonography were normal for gestational age. Echocardiography demonstrated structurally normal cardiac anatomy with suprasystemic pulmonary artery pressures, right-to-left shunting at the patent ductus arteriosus and patent foramen ovale, severe right ventricular pressure overload, and significant biventricular systolic dysfunction. These findings were consistent with severe pulmonary vascular disease and PPHN.

**Figure 2:**
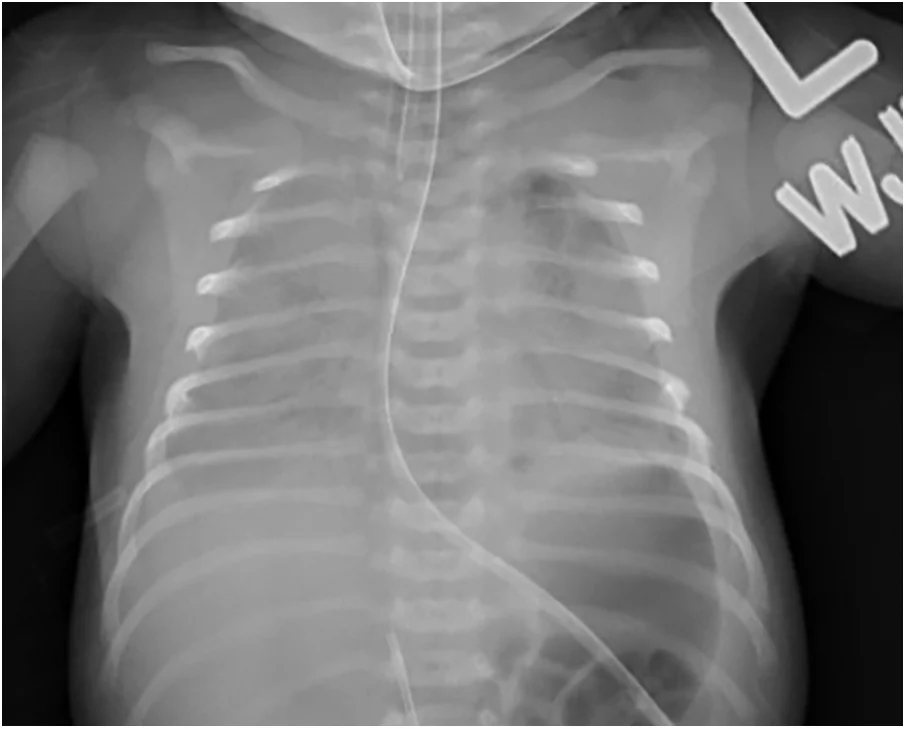
Initial chest radiograph of Case 2 shows markedly low lung volumes and poor aeration consistent with pulmonary hypoplasia.

Despite treatment with inhaled nitric oxide, inotropic support, and vasoactive medications, oxygenation and ventilation remained critically impaired. Serial echocardiograms demonstrated progressive deterioration in ventricular function without improvement. Despite maximal medical management, the infant experienced ongoing cardiorespiratory deterioration. After extensive discussions regarding prognosis and treatment limitations, the family elected for comfort-focused care. Life-sustaining therapies were withdrawn, and the infant died at 9 h of life.

## Postmortem findings

### Gross findings

Complete autopsies were performed in both cases. Gross examination demonstrated markedly reduced lung size and weight with firm, poorly expanded parenchyma. Combined lung weights were 30 g in Case 1 (expected 59.6 g) and 25 g in Case 2 (expected 54.1 g), resulting in significantly reduced lung-to-body-weight ratios consistent with severe bilateral pulmonary hypoplasia. No major extra-cardiopulmonary anomalies were identified.

### Histopathologic findings

Histopathologic examination revealed severe disruption of distal lung development in both infants, characterized by markedly reduced alveolarization, simplified airspaces, diminished radial alveolar counts, immature distal airspaces, and poor acinar development. The lungs were composed predominantly of bronchiolar structures with only rare respiratory bronchioles, alveolar ducts, and primitive alveolar saccules, producing a pattern closely resembling acinar dysplasia.

Occasional bronchioles and alveolar spaces contained mucin, squamous debris, and rare meconium-laden histiocytes. The interstitium was immature with focal collections of primitive spindle-shaped mesenchymal cells. Evaluation of the pulmonary vasculature demonstrated patent arteries without evidence of thrombi or significant vascular remodeling, and there was no evidence of alveolar capillary dysplasia or misalignment of pulmonary veins. Cartilaginous rings of the trachea and major bronchi were normally developed. No autopsy evidence of central nervous system abnormalities, prolonged anhydramnios, skeletal dysplasia, congenital neuromuscular disease, or other recognized causes of secondary pulmonary hypoplasia was identified.

### Summary interpretation

Overall, the findings were consistent with a severe developmental lung disorder within the spectrum of *TBX4*-associated developmental lung disease, exhibiting pathologic features most closely resembling acinar dysplasia with severe pulmonary hypoplasia.

## Genetic testing

Chromosomal microarray analysis (CMA) was performed in both cases. In Case 1, no clinically significant copy number abnormalities were identified. In Case 2, CMA detected a duplication involving chromosome 3p26.3–p26.2 encompassing the *CNTN4* gene (OMIM #607280). This copy number variant was classified as a variant of uncertain significance and was not considered sufficient to explain the severe neonatal pulmonary phenotype.

Given the recurrent occurrence of neonatal-lethal respiratory failure in siblings, whole-exome sequencing (WES) was performed in Case 2 and identified a novel heterozygous missense variant in *TBX4*, c.741G>C (p.Lys247Asn), classified as likely pathogenic. Parental segregation analysis confirmed paternal inheritance, consistent with autosomal dominant transmission. The father was clinically unaffected, suggesting incomplete penetrance and variable expressivity, which are well-recognized features of *TBX4*-related disorders. The pulmonary architecture demonstrated findings consistent with a developmental lung disorder within the spectrum of *TBX4*-associated pulmonary disease. No significant inflammatory process or alternative structural explanation for the severe respiratory failure was identified.

The parents opted for *in vitro* fertilization with preimplantation genetic testing for monogenic disease (PGT-M). Ten embryos were analyzed: seven carried the familial *TBX4* c.741G>C (p.Lys247Asn) variant, two demonstrated additional chromosomal abnormalities involving chromosomes 3 and 7 that rendered them unsuitable for transfer, and one embryo required repeat testing because of an inconclusive result. Consequently, no embryos were selected for implantation.

## Discussion

Primary pulmonary hypoplasia is a rare congenital disorder with an estimated incidence of 9–11 per 10,000 live births and mortality rates approaching 71%–95% in severe cases (1–3). It is characterized by abnormal development of the pulmonary airways, alveoli, and vasculature, leading to profound respiratory insufficiency. Histological diagnosis relies on lung weight-to-birth weight ratio and radial alveolar count (RAC) criteria (4). Both infants in our report demonstrated markedly reduced lung weight-to-body weight ratios and decreased RAC, consistent with severe pulmonary hypoplasia. Table 1 summarizes the case report of primary pulmonary hypoplasia. Primary genetic causes of PH are being increasingly recognized, particularly among infants with recurrent familial disease (6).

**Table 1:** Summary of reported cases with primary congenital pulmonary hypoplasia.

Reporter and year	Case no.	Mortality	Lung-to-body weight ratio (%)	RAC^a^
Mendelsohn and Hutchins (1977) (25)	1	1/1	0.21	Not checked
Boylan et al. (1977) (26)	2	2/2	1.15	Not checked
Swischuk et al. (1979) (27)	8	6/8	0.98	Reduced
Langer and Kaufmann (1986) (28)	9	8/9	2.14	Reduced
Chambers (1991) (29)	1	1/1	0.85	Not checked
Frey et al. (1994) (30)	3	3/3	0.57	Normal
Hamel (1995) (31)	2	2/2	0.35	Not checked
Moerman et al. (1998) (32)	2	2/2	0.69	Not checked
Green et al. (1999) (33)	1	1/1	Not checked	Not checked
Odd et al. (2003) (34)	1	1/1	2.56	Not checked
Hsu et al. (2010) (35)	1	1/1	0.69	Reduced
This case (2017, 2019)	2	2/2	0.92	Reduced

RAC, radial alveoli count. Adapted from Hsu et al. (35).

a RAC: A line was dropped from the center of each respiratory bronchiole (bronchioles only partially lined with epithelium) to the nearest connective tissue septum at a right angle to the epithelium. The number of alveolar septa cut by this line was counted.

In the literature review of the reported cases with congenital primary pulmonary hypoplasia from 1977 to 2017, mortality was high and only 3/30 infants survived to discharge from the NICU (Table 1).

The T-box family of genes (*TBX1–5*) is highly expressed during both early and later stages of embryogenesis. *TBX4*, a member of this family, plays a critical role in limb and lung development (7–9). Heterozygous variants in *TBX4* have been associated with small patella syndrome and/or PAH, with onset occurring in childhood or adulthood (9–11). Both *de novo* and inherited *TBX4* variants have been documented (9, 12). In addition, microdeletions involving chromosomal region 17q23.2, which includes *TBX4* and other genes, have been reported in individuals with skeletal anomalies and/or PAH, often accompanied by features such as intellectual disability, global developmental delay, congenital heart defects, microcephaly, postnatal growth restriction, facial dysmorphism, and hearing loss (9, 12, 13). Duplications of 17q23.2 involving *TBX4* have also been linked to clubfoot, with or without additional anomalies (6). Furthermore, a *de novo* frameshift variant and a 2.2 Mb deletion on chromosome 17 involving *TBX4* have been described in two unrelated individuals with neonatal lung disease who were also heterozygous for the same ABCA3 missense variant (14). Supporting its role in neonatal lung development, heterozygous deletions of *TBX4* and *de novo* missense variants have been associated with acinar dysplasia in deceased neonates presenting with severe lung disorders (15).

Recent studies have established *TBX4* as an important cause of developmental lung disease. Neonatal presentations are being increasingly recognized and include acinar dysplasia, congenital alveolar dysplasia, pulmonary hypoplasia, and severe pulmonary vascular abnormalities (6, 14–16). Galambos et al. (16) demonstrated that *TBX4*-related disorders encompass a broad histopathological spectrum involving both distal lung development and pulmonary vascular morphogenesis. The severe neonatal respiratory failure observed in our patients is consistent with these previously reported phenotypes.

Homozygous mutations of the *TBX4* gene have been implicated in posterior amelia with pelvis and pulmonary hypoplasia syndrome, while heterozygous mutations of the *TBX4* gene may exhibit the milder ischio-coxo-podo-patellar (“small patella”) syndrome with or without pulmonary arterial hypertension (17, 18). Notably, in both cases described herein, the lower extremities were anatomically normal at birth, and pelvic abnormalities cannot be determined until ossification is complete.

A novel heterozygous frameshift variant in the *TBX4* gene (c.1160delC) has been reported as pathogenic and associated with persistent pulmonary hypertension (19). In addition, an autosomal dominant *TBX4* mutation has been linked to variable degrees of pulmonary hypertension within affected families, underscoring the phenotypic heterogeneity of this disorder (18). Experimental models demonstrate that postnatal disruption of *TBX4* impairs lung function by reducing alveolar growth, downregulating pro-angiogenic mediators, and limiting vascular development, ultimately contributing to pulmonary hypertension (19–21). Consistent with these findings, both cases in our report exhibited severe pulmonary arterial hypertension secondary to pulmonary vascular and parenchymal hypoplasia, which proved refractory to conventional therapy and iNO.

In our case, WES identified a heterozygous *TBX4* missense variant—p.Lys247Asn (AAG>AAC), c.741 G>C in exon 6 of the *TBX4* gene. This variant has not been previously reported in the published literature and was classified as likely pathogenic. Paternal inheritance of the autosomal dominant *TBX4* variant was confirmed. The substituted residue is located within the highly conserved T-box DNA-binding domain of *TBX4*, a critical functional domain responsible for sequence-specific DNA binding and transcriptional regulation of downstream target genes. *In silico* prediction supports a deleterious effect of the amino-acid substitution. Importantly, in a multicenter study of 137 individuals with monoallelic *TBX4* variants, genotype–phenotype analysis by Prapa et al. (22) demonstrated that *TBX4* variants involving the T-box and nuclear localization domains were associated with earlier clinical presentation and an increased incidence of interstitial lung disease and pulmonary arterial hypertension; variants affecting the T-box domain were also associated with shorter event-free survival, although this association was influenced by age at diagnosis. These observations provide additional biological and clinical context supporting the potential pathogenic relevance of the p.Lys247Asn variant in the setting of the severe neonatal phenotype observed in both siblings. Nevertheless, functional studies of this specific variant were not performed, and its classification should therefore be interpreted in conjunction with the totality of available genetic, clinical, and pathological evidence. The recurrent occurrence of an identical severe phenotype in two siblings, combined with the absence of other clinically significant abnormalities on evaluation, strongly supports the likely pathogenic role of this variant. Further research is warranted to elucidate its functional consequences, penetrance, genotype–phenotype correlations, and potential therapeutic targets.

An important feature of our report is the identification of the same *TBX4* variant in an apparently unaffected father. *TBX4*-associated disorders are characterized by marked phenotypic heterogeneity, variable expressivity, and incomplete penetrance. Previous reports have described asymptomatic carriers, isolated pulmonary hypertension, small patella syndrome, and lethal neonatal lung disease occurring within the same family (23). Moreover, previous studies have demonstrated marked intrafamilial variability among individuals carrying the same *TBX4* variant, ranging from unaffected carriers to severe pulmonary and skeletal disease, including lethal neonatal lung disease (6). Haploinsufficiency of the *TBX4* gene resulting from heterozygous deletion has been reported to disrupt fetal lung development and cause congenital alveolar dysplasia (24).

The phenotypic heterogeneity observed in the family described in this report may similarly reflect the influence of additional genetic modifiers or “second-hit” variants that further reduce *TBX4* activity during critical developmental stages. Given the role of *TBX4* as a developmental transcription factor, disease expression is also likely influenced by dosage- and tissue-dependent effects, with differences in residual *TBX4* function contributing to variable involvement of the lungs, pulmonary vasculature, and skeletal system. Epigenetic and environmental factors may further modulate disease expression. Although these mechanisms remain speculative, they provide plausible explanations for the variable clinical manifestations observed among carriers of the same *TBX4* variant (6, 23).

The absence of clinically apparent disease in the father may reflect incomplete penetrance or reduced phenotypic expression, although subclinical manifestations cannot be excluded. Because additional modifier variants were not assessed, these mechanisms remain speculative and should be considered potential rather than definitive explanations for the observed phenotypic variability.

Prenatal diagnosis of *TBX4*-associated developmental lung disease remains challenging because standard fetal ultrasonography may appear normal until late gestation. In families with known pathogenic variants, serial fetal imaging, fetal MRI, and specialized Doppler assessment of the fetal pulmonary circulation may provide additional information regarding lung development. Genetic counseling should include discussion of prenatal diagnosis through chorionic villus sampling or amniocentesis and preimplantation genetic testing for monogenic disorders when available.

## Conclusion

We report two siblings with recurrent lethal neonatal developmental lung disease caused by a novel heterozygous *TBX4* variant inherited from an apparently unaffected father, which was identified via WES in the second infant. Although the specific variant was not functionally characterized and molecular confirmation was available only in the second infant, the recurrent and highly concordant clinicopathologic phenotype in the two siblings, together with the location of the variant within the critical T-box domain and established *TBX4* genotype–phenotype correlations, supports its likely contribution to the observed developmental lung disease, which may present with profound respiratory failure and pulmonary hypertension even when routine prenatal imaging is unremarkable. Recognition of incomplete penetrance and variable expressivity is essential for accurate genetic counseling, recurrence risk assessment, prenatal diagnosis, and reproductive planning in affected families.

## Data Availability

The raw data supporting the conclusions of this article will be made available by the authors, without undue reservation.
